# (1*Z*)-1-[(2*E*)-3-(4-Bromo­phen­yl)-1-(4-fluoro­phen­yl)prop-2-en-1-yl­idene]-2-(2,4-dinitro­phen­yl)hydrazine

**DOI:** 10.1107/S1600536812027328

**Published:** 2012-06-23

**Authors:** Rajni Kant, Vivek K. Gupta, Kamini Kapoor, M. Sapnakumari, B. K. Sarojini, B. Narayana

**Affiliations:** a X-ray Crystallography Laboratory, Post-Graduate Department of Physics & Electronics, University of Jammu, Jammu Tawi 180 006, India; b Department of Studies in Chemistry, Mangalore University, Mangalagangotri 574 199, India; c Department of Chemistry, P.A. College of Engineering, Nadupadavu, Mangalore 574 153, India

## Abstract

In the title mol­ecule, C_21_H_14_BrFN_4_O_4_, the mean planes of the two nitro groups form dihedral angles of 3.1 (2) and 7.1 (5)° with the benzene ring to which they are attached. The dinitro-substituted ring forms dihedral angles of 8.6 (2) and 71.9 (2)° with the bromo- and fluoro-substituted benzene rings, respectively. The dihedral angle between the bromo- and fluoro-substituted benzene rings is 80.6 (2)°. There is an intra­molecular N—H⋯O hydrogen bond. In the crystal, pairs of weak C—H⋯O hydrogen bonds form inversion dimers. In addition, π–π stacking inter­actions between the bromo- and dinitro-substituted rings [centroid–centroid separation = 3.768 (2) Å] are observed.

## Related literature

For applications of hydrazone derivatives, see: Rollas *et al.* (2007 ▶); Singh *et al.* (1982 ▶). For the synthesis, see: Jasinski *et al.* (2010 ▶). For a related structure, see: Yin *et al.* (2009 ▶).
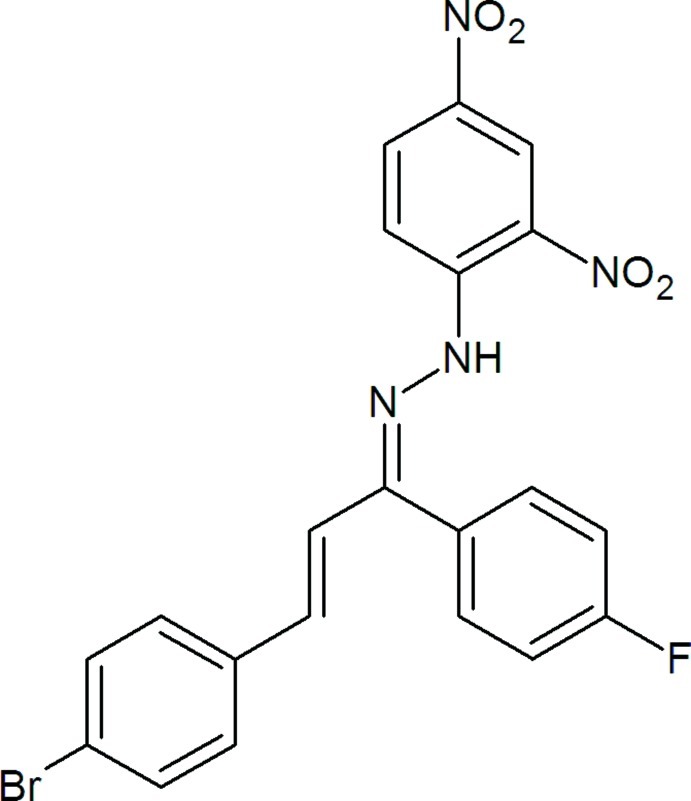


## Experimental

### 

#### Crystal data

C_21_H_14_BrFN_4_O_4_
*M* *_r_* = 485.27
Monoclinic, 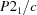

*a* = 15.0738 (12) Å
*b* = 10.6511 (5) Å
*c* = 14.3353 (8) Å
β = 116.010 (9)°
*V* = 2068.5 (2) Å^3^
*Z* = 4
Mo *K*α radiation
μ = 2.03 mm^−1^
*T* = 293 K
0.3 × 0.2 × 0.1 mm

#### Data collection

Oxford Diffraction Xcalibur Sapphire3 diffractometer
Absorption correction: multi-scan (*CrysAlis PRO*; Oxford Diffraction, 2010 ▶) *T*_min_ = 0.889, *T*_max_ = 1.000
15619 measured reflections
4058 independent reflections
2232 reflections with *I* > 2σ(*I*)
*R*_int_ = 0.045

#### Refinement

*R*[*F* ^2^ > 2σ(*F* ^2^)] = 0.051
*wR*(*F* ^2^) = 0.141
*S* = 1.01
4058 reflections
280 parameters
H-atom parameters constrained
Δρ_max_ = 0.30 e Å^−3^
Δρ_min_ = −0.40 e Å^−3^

### 

Data collection: *CrysAlis PRO* (Oxford Diffraction, 2010 ▶); cell refinement: *CrysAlis PRO*; data reduction: *CrysAlis PRO*; program(s) used to solve structure: *SHELXS97* (Sheldrick, 2008 ▶); program(s) used to refine structure: *SHELXL97* (Sheldrick, 2008 ▶); molecular graphics: *ORTEP-3* (Farrugia, 1997 ▶); software used to prepare material for publication: *PLATON* (Spek, 2009 ▶).

## Supplementary Material

Crystal structure: contains datablock(s) I, global. DOI: 10.1107/S1600536812027328/lh5491sup1.cif

Structure factors: contains datablock(s) I. DOI: 10.1107/S1600536812027328/lh5491Isup2.hkl

Supplementary material file. DOI: 10.1107/S1600536812027328/lh5491Isup3.cml

Additional supplementary materials:  crystallographic information; 3D view; checkCIF report

## Figures and Tables

**Table 1:** Hydrogen-bond geometry (Å, °)

*D*—H⋯*A*	*D*—H	H⋯*A*	*D*⋯*A*	*D*—H⋯*A*
N2—H21⋯O1	0.86	1.95	2.584 (4)	130
C11—H11⋯O4^i^	0.93	2.45	3.316 (7)	154

Symmetry code: (i) 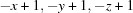.

**Fig. 1.**
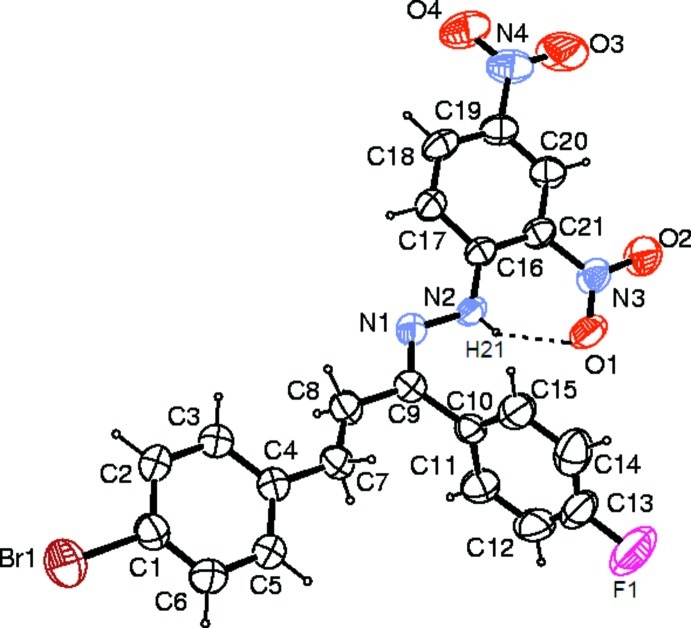
The molecular structure of (I) with ellipsoids drawn at the 40% probability level. H atoms are shown as small spheres of arbitrary radii. The dashed line indicates a hydrogen bond.

**Fig. 2.**
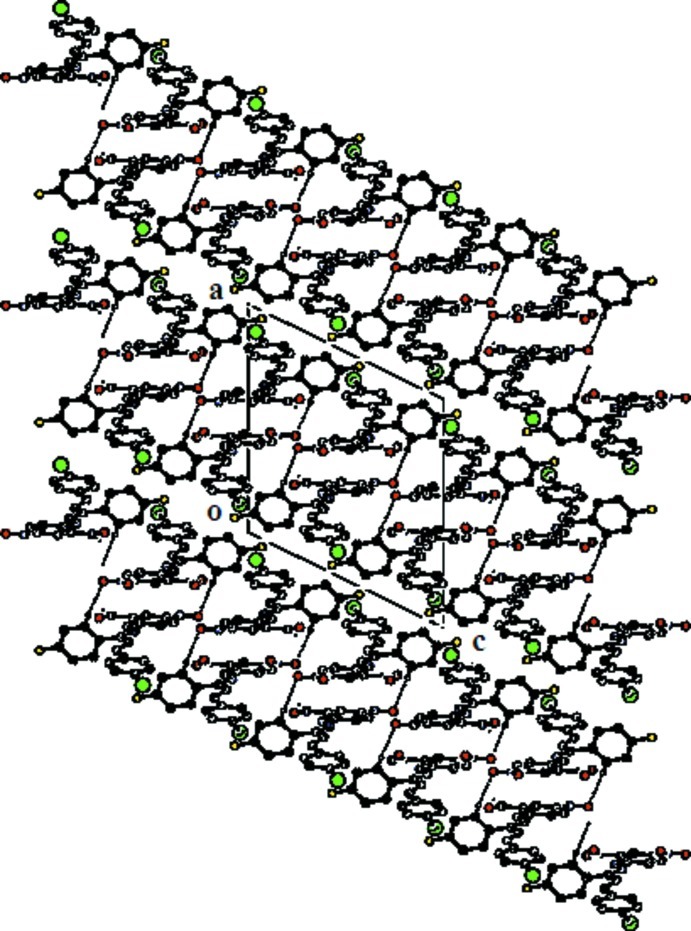
The packing arrangement of molecules viewed along the b axis. The broken lines show intermolecular C—H···O interactions.

## supplementary crystallographic information

### Comment

Hydrazone derivatives are important biologically active compounds which have received attention from the synthetic community (Rollas *et al.*, 2007). Hydrazone derivatives are also used as analytical reagents (Singh *et al.*, 1982). The crystal structure of 1-(but-2-enylidene)-2-(2-nitrophenyl)hydrazine has been reported (Yin *et al.*, 2009). In order to prepare a pyrazoline derivative, (2E)-3-(4-bromophenyl) -1-(4-fluorophenyl)prop-2-en-1-one was reacted with 2,4-dinitrophenyl hydrazine as for the method of Jasinski *et al.* (2010). But, instead of a pyrazoline derivative a 2,4-dintrophenylhydrazone compound (I) was obtained and its crystal structure is reported herein.

In (I) (Fig. 1), all bond lengths and angles are normal and correspond to those which are related in a reported structure (Yin *et al.*, 2009). The two nitro groups form dihedral angles of 3.1 (2) and 7.1 (5)° with the C16-C21 ring. The dinitro substituted ring (C16-C21) forms dihedral angles of 8.6 (2)° and 71.9 (2) ° with bromo (C1-C6) and fluoro (C10-C15) substituted benzene rings, respectively. The dihedral angle between the bromo and fluoro substituted benzene rings is 80.6 (2)°. There is an intramolecular N—H···O hydrogen bond and in the crystal, pairs of weak C—H···O hydrogen bonds form inversion dimers (Table 1, Fig. 2). In addition, π–π stacking interactions between the bromophenyl ring and dinitro phenyl ring are observed [centroid separation = 3.768 (2) Å, interplanar spacing =3.410 Å, centroid shift = 1.60 Å, Symmetry = *x*, 1 + *y*, *z*].

### Experimental

A mixture of (2E)-3-(4-bromophenyl)-1-(4-fluorophenyl)prop-2-en-1-one (3.05 g, 0.01 mol) and 2,4-dinitrophenylhydrazine (1.98 g, 0.01 mol) in 50 ml of glacial acetic acid was refluxed for 6 hrs. The reaction mixture was cooled to produce red crystals (m.p. 414–416 K). X-ray quality crystals were obtained by slow evaporation of an acetic acid solution of (I) at room temperature.

### Refinement

All H atoms were positioned geometrically and were treated as riding on their parent C/N atoms, with N—H distance of 0.86 Å and C—H distances of 0.93 Å and with *U*_iso_(H) = 1.2*U*_eq_(C,N).

### Crystal data

**Table:**

C_21_H_14_BrFN_4_O_4_	*F*(000) = 976
*M**_r_* = 485.27	*D*_x_ = 1.558 Mg m^−^^3^
Monoclinic, *P*2_1_/*c*	Melting point = 416–414 K
Hall symbol: -P 2ybc	Mo *K*α radiation, λ = 0.71073 Å
*a* = 15.0738 (12) Å	Cell parameters from 3762 reflections
*b* = 10.6511 (5) Å	θ = 3.6–29.0°
*c* = 14.3353 (8) Å	µ = 2.03 mm^−^^1^
β = 116.010 (9)°	*T* = 293 K
*V* = 2068.5 (2) Å^3^	Plate, red
*Z* = 4	0.3 × 0.2 × 0.1 mm

### Data collection

**Table:**

Oxford Diffraction Xcalibur Sapphire3 diffractometer	4058 independent reflections
Radiation source: fine-focus sealed tube	2232 reflections with *I* > 2σ(*I*)
Graphite monochromator	*R*_int_ = 0.045
Detector resolution: 16.1049 pixels mm^-1^	θ_max_ = 26.0°, θ_min_ = 3.6°
ω scans	*h* = −18→18
Absorption correction: multi-scan (*CrysAlis PRO*; Oxford Diffraction, 2010)	*k* = −13→13
*T*_min_ = 0.889, *T*_max_ = 1.000	*l* = −17→17
15619 measured reflections	

### Refinement

**Table:**

Refinement on *F*^2^	Primary atom site location: structure-invariant direct methods
Least-squares matrix: full	Secondary atom site location: difference Fourier map
*R*[*F*^2^ > 2σ(*F*^2^)] = 0.051	Hydrogen site location: inferred from neighbouring sites
*wR*(*F*^2^) = 0.141	H-atom parameters constrained
*S* = 1.01	*w* = 1/[σ^2^(*F*_o_^2^) + (0.0533*P*)^2^ + 0.8232*P*] where *P* = (*F*_o_^2^ + 2*F*_c_^2^)/3
4058 reflections	(Δ/σ)_max_ = 0.001
280 parameters	Δρ_max_ = 0.30 e Å^−^^3^
0 restraints	Δρ_min_ = −0.40 e Å^−^^3^

### Special details

**Table:**

Experimental. *CrysAlis PRO*, Oxford Diffraction Ltd., Version 1.171.34.40 (release 27–08-2010 CrysAlis171. NET) (compiled Aug 27 2010,11:50:40) Empirical absorption correction using spherical harmonics, implemented in SCALE3 ABSPACK scaling algorithm.
Geometry. All e.s.d.'s (except the e.s.d. in the dihedral angle between two l.s. planes) are estimated using the full covariance matrix. The cell e.s.d.'s are taken into account individually in the estimation of e.s.d.'s in distances, angles and torsion angles; correlations between e.s.d.'s in cell parameters are only used when they are defined by crystal symmetry. An approximate (isotropic) treatment of cell e.s.d.'s is used for estimating e.s.d.'s involving l.s. planes.
Refinement. Refinement of *F*^2^ against ALL reflections. The weighted *R*-factor *wR* and goodness of fit *S* are based on *F*^2^, conventional *R*-factors *R* are based on *F*, with *F* set to zero for negative *F*^2^. The threshold expression of *F*^2^ > σ(*F*^2^) is used only for calculating *R*-factors(gt) *etc*. and is not relevant to the choice of reflections for refinement. *R*-factors based on *F*^2^ are statistically about twice as large as those based on *F*, and *R*- factors based on ALL data will be even larger.

### Fractional atomic coordinates and isotropic or equivalent isotropic displacement parameters (Å^2^)

**Table:**

	*x*	*y*	*z*	*U*_iso_*/*U*_eq_	
Br1	0.10475 (5)	1.47051 (5)	0.45912 (4)	0.1080 (3)	
F1	0.0359 (3)	0.6564 (3)	−0.06938 (19)	0.1523 (14)	
N2	0.3279 (2)	0.5513 (3)	0.3799 (2)	0.0541 (7)	
H21	0.3030	0.5395	0.3139	0.065*	
O1	0.3072 (2)	0.3913 (3)	0.23628 (19)	0.0930 (10)	
O2	0.3459 (3)	0.1980 (3)	0.2728 (2)	0.0946 (10)	
O3	0.4846 (3)	0.0425 (4)	0.6152 (3)	0.1316 (16)	
O4	0.5243 (3)	0.1687 (4)	0.7432 (3)	0.1200 (13)	
N3	0.3419 (3)	0.3052 (4)	0.2993 (2)	0.0700 (9)	
N1	0.3238 (2)	0.6688 (3)	0.4176 (2)	0.0581 (8)	
N4	0.4898 (3)	0.1471 (5)	0.6501 (4)	0.0932 (12)	
C1	0.1384 (3)	1.3158 (4)	0.4190 (3)	0.0624 (10)	
C2	0.1915 (3)	1.2297 (4)	0.4951 (3)	0.0620 (10)	
H2	0.2122	1.2492	0.5648	0.074*	
C3	0.2135 (3)	1.1146 (4)	0.4666 (3)	0.0615 (10)	
H3	0.2490	1.0559	0.5173	0.074*	
C4	0.1829 (3)	1.0855 (3)	0.3623 (3)	0.0574 (9)	
C5	0.1301 (3)	1.1750 (4)	0.2885 (3)	0.0655 (10)	
H5	0.1090	1.1564	0.2186	0.079*	
C6	0.1080 (3)	1.2911 (4)	0.3162 (3)	0.0692 (11)	
H6	0.0732	1.3507	0.2660	0.083*	
C7	0.2021 (3)	0.9630 (3)	0.3285 (3)	0.0621 (10)	
H7	0.1672	0.9451	0.2581	0.074*	
C8	0.2633 (3)	0.8747 (3)	0.3863 (3)	0.0613 (10)	
H8	0.3046	0.8936	0.4553	0.074*	
C9	0.2700 (3)	0.7506 (3)	0.3489 (3)	0.0560 (9)	
C10	0.2108 (3)	0.7207 (3)	0.2368 (3)	0.0523 (9)	
C11	0.2375 (4)	0.7628 (4)	0.1622 (3)	0.0780 (12)	
H11	0.2958	0.8080	0.1816	0.094*	
C12	0.1792 (5)	0.7388 (5)	0.0598 (4)	0.0965 (17)	
H12	0.1981	0.7659	0.0095	0.116*	
C13	0.0950 (5)	0.6765 (5)	0.0324 (3)	0.0895 (16)	
C14	0.0659 (3)	0.6315 (5)	0.1030 (4)	0.0905 (14)	
H14	0.0074	0.5864	0.0822	0.109*	
C15	0.1253 (3)	0.6545 (4)	0.2068 (3)	0.0695 (11)	
H15	0.1068	0.6246	0.2566	0.083*	
C16	0.3702 (2)	0.4545 (3)	0.4448 (2)	0.0495 (8)	
C17	0.4066 (3)	0.4703 (4)	0.5530 (2)	0.0565 (9)	
H17	0.4031	0.5488	0.5797	0.068*	
C18	0.4465 (3)	0.3722 (4)	0.6188 (3)	0.0643 (11)	
H18	0.4705	0.3840	0.6900	0.077*	
C19	0.4517 (3)	0.2545 (4)	0.5802 (3)	0.0614 (10)	
C20	0.4187 (3)	0.2341 (4)	0.4765 (3)	0.0614 (10)	
H20	0.4231	0.1549	0.4516	0.074*	
C21	0.3786 (2)	0.3336 (4)	0.4092 (2)	0.0537 (9)	

### Atomic displacement parameters (Å^2^)

**Table:**

	*U*^11^	*U*^22^	*U*^33^	*U*^12^	*U*^13^	*U*^23^
Br1	0.1532 (6)	0.0716 (4)	0.1080 (4)	0.0292 (3)	0.0652 (4)	−0.0073 (3)
F1	0.197 (3)	0.147 (3)	0.0548 (16)	0.047 (3)	0.0025 (18)	−0.0091 (17)
N2	0.0666 (19)	0.0520 (18)	0.0393 (15)	0.0087 (15)	0.0193 (14)	0.0017 (14)
O1	0.145 (3)	0.077 (2)	0.0458 (15)	0.027 (2)	0.0320 (17)	0.0036 (16)
O2	0.142 (3)	0.073 (2)	0.079 (2)	0.022 (2)	0.0581 (19)	−0.0100 (16)
O3	0.181 (4)	0.087 (3)	0.122 (3)	0.061 (3)	0.062 (3)	0.047 (2)
O4	0.114 (3)	0.140 (3)	0.074 (2)	0.031 (2)	0.0118 (19)	0.046 (2)
N3	0.085 (2)	0.072 (2)	0.058 (2)	0.015 (2)	0.0360 (18)	−0.0049 (19)
N1	0.0656 (19)	0.0526 (18)	0.0520 (17)	0.0019 (16)	0.0219 (15)	−0.0028 (16)
N4	0.084 (3)	0.099 (3)	0.086 (3)	0.026 (3)	0.028 (2)	0.040 (3)
C1	0.072 (3)	0.051 (2)	0.068 (2)	0.001 (2)	0.034 (2)	−0.003 (2)
C2	0.073 (3)	0.060 (2)	0.050 (2)	−0.002 (2)	0.025 (2)	−0.0078 (19)
C3	0.070 (3)	0.054 (2)	0.054 (2)	0.002 (2)	0.0223 (19)	0.0022 (19)
C4	0.066 (2)	0.049 (2)	0.054 (2)	−0.0019 (19)	0.0235 (19)	−0.0034 (18)
C5	0.084 (3)	0.055 (2)	0.051 (2)	−0.001 (2)	0.024 (2)	−0.0016 (19)
C6	0.081 (3)	0.062 (3)	0.057 (2)	0.010 (2)	0.023 (2)	0.005 (2)
C7	0.079 (3)	0.051 (2)	0.055 (2)	−0.004 (2)	0.028 (2)	−0.0027 (19)
C8	0.072 (3)	0.050 (2)	0.056 (2)	−0.004 (2)	0.022 (2)	−0.0055 (19)
C9	0.062 (2)	0.051 (2)	0.057 (2)	−0.0016 (19)	0.0279 (19)	0.0011 (19)
C10	0.066 (2)	0.0431 (19)	0.050 (2)	0.0094 (18)	0.0277 (19)	0.0034 (16)
C11	0.102 (3)	0.075 (3)	0.069 (3)	−0.005 (3)	0.049 (3)	0.003 (2)
C12	0.165 (6)	0.078 (3)	0.064 (3)	0.017 (4)	0.066 (4)	0.011 (3)
C13	0.120 (4)	0.080 (3)	0.042 (3)	0.036 (3)	0.011 (3)	−0.003 (2)
C14	0.073 (3)	0.096 (4)	0.081 (3)	0.009 (3)	0.013 (3)	−0.014 (3)
C15	0.072 (3)	0.077 (3)	0.058 (2)	0.003 (2)	0.026 (2)	0.001 (2)
C16	0.045 (2)	0.057 (2)	0.0428 (19)	0.0024 (17)	0.0162 (16)	0.0023 (17)
C17	0.058 (2)	0.061 (2)	0.046 (2)	−0.0004 (19)	0.0180 (18)	−0.0017 (18)
C18	0.051 (2)	0.091 (3)	0.0410 (19)	−0.002 (2)	0.0110 (17)	0.006 (2)
C19	0.050 (2)	0.071 (3)	0.058 (2)	0.013 (2)	0.0180 (19)	0.019 (2)
C20	0.058 (2)	0.061 (2)	0.065 (2)	0.0100 (19)	0.0274 (19)	0.007 (2)
C21	0.054 (2)	0.062 (2)	0.047 (2)	0.0077 (19)	0.0236 (17)	0.0017 (18)

### Geometric parameters (Å, º)

**Table:**

Br1—C1	1.886 (4)	C7—H7	0.9300
F1—C13	1.352 (5)	C8—C9	1.447 (5)
N2—C16	1.347 (4)	C8—H8	0.9300
N2—N1	1.376 (4)	C9—C10	1.491 (5)
N2—H21	0.8600	C10—C15	1.363 (5)
O1—N3	1.231 (4)	C10—C11	1.372 (5)
O2—N3	1.213 (4)	C11—C12	1.365 (6)
O3—N4	1.209 (5)	C11—H11	0.9300
O4—N4	1.223 (5)	C12—C13	1.330 (7)
N3—C21	1.455 (4)	C12—H12	0.9300
N1—C9	1.298 (4)	C13—C14	1.356 (7)
N4—C19	1.463 (5)	C14—C15	1.381 (5)
C1—C6	1.363 (5)	C14—H14	0.9300
C1—C2	1.381 (5)	C15—H15	0.9300
C2—C3	1.378 (5)	C16—C21	1.411 (5)
C2—H2	0.9300	C16—C17	1.411 (4)
C3—C4	1.393 (5)	C17—C18	1.359 (5)
C3—H3	0.9300	C17—H17	0.9300
C4—C5	1.386 (5)	C18—C19	1.386 (5)
C4—C7	1.464 (5)	C18—H18	0.9300
C5—C6	1.384 (5)	C19—C20	1.361 (5)
C5—H5	0.9300	C20—C21	1.381 (5)
C6—H6	0.9300	C20—H20	0.9300
C7—C8	1.324 (5)		
			
C16—N2—N1	120.9 (3)	C15—C10—C11	118.9 (4)
C16—N2—H21	119.5	C15—C10—C9	119.1 (3)
N1—N2—H21	119.5	C11—C10—C9	121.9 (4)
O2—N3—O1	122.3 (3)	C12—C11—C10	120.4 (5)
O2—N3—C21	119.1 (3)	C12—C11—H11	119.8
O1—N3—C21	118.6 (3)	C10—C11—H11	119.8
C9—N1—N2	115.6 (3)	C13—C12—C11	119.6 (4)
O3—N4—O4	123.0 (4)	C13—C12—H12	120.2
O3—N4—C19	120.1 (4)	C11—C12—H12	120.2
O4—N4—C19	116.9 (5)	C12—C13—F1	119.3 (6)
C6—C1—C2	122.0 (4)	C12—C13—C14	122.3 (4)
C6—C1—Br1	119.4 (3)	F1—C13—C14	118.3 (6)
C2—C1—Br1	118.6 (3)	C13—C14—C15	118.2 (5)
C3—C2—C1	119.2 (3)	C13—C14—H14	120.9
C3—C2—H2	120.4	C15—C14—H14	120.9
C1—C2—H2	120.4	C10—C15—C14	120.6 (4)
C2—C3—C4	120.5 (3)	C10—C15—H15	119.7
C2—C3—H3	119.8	C14—C15—H15	119.7
C4—C3—H3	119.8	N2—C16—C21	122.7 (3)
C5—C4—C3	118.4 (3)	N2—C16—C17	120.4 (3)
C5—C4—C7	119.4 (3)	C21—C16—C17	116.9 (3)
C3—C4—C7	122.2 (3)	C18—C17—C16	120.8 (4)
C6—C5—C4	121.7 (3)	C18—C17—H17	119.6
C6—C5—H5	119.2	C16—C17—H17	119.6
C4—C5—H5	119.2	C17—C18—C19	120.3 (3)
C1—C6—C5	118.3 (4)	C17—C18—H18	119.9
C1—C6—H6	120.9	C19—C18—H18	119.9
C5—C6—H6	120.9	C20—C19—C18	121.5 (3)
C8—C7—C4	127.6 (3)	C20—C19—N4	118.0 (4)
C8—C7—H7	116.2	C18—C19—N4	120.5 (4)
C4—C7—H7	116.2	C19—C20—C21	118.6 (4)
C7—C8—C9	124.0 (3)	C19—C20—H20	120.7
C7—C8—H8	118.0	C21—C20—H20	120.7
C9—C8—H8	118.0	C20—C21—C16	122.0 (3)
N1—C9—C8	116.9 (3)	C20—C21—N3	116.1 (3)
N1—C9—C10	123.7 (3)	C16—C21—N3	121.9 (3)
C8—C9—C10	119.3 (3)		
			
C16—N2—N1—C9	−171.1 (3)	F1—C13—C14—C15	−178.6 (4)
C6—C1—C2—C3	−0.8 (6)	C11—C10—C15—C14	−0.8 (6)
Br1—C1—C2—C3	177.7 (3)	C9—C10—C15—C14	176.3 (4)
C1—C2—C3—C4	0.3 (6)	C13—C14—C15—C10	0.0 (6)
C2—C3—C4—C5	0.0 (6)	N1—N2—C16—C21	−178.0 (3)
C2—C3—C4—C7	−178.1 (4)	N1—N2—C16—C17	3.5 (5)
C3—C4—C5—C6	0.3 (6)	N2—C16—C17—C18	177.9 (3)
C7—C4—C5—C6	178.4 (4)	C21—C16—C17—C18	−0.7 (5)
C2—C1—C6—C5	1.1 (6)	C16—C17—C18—C19	−0.3 (5)
Br1—C1—C6—C5	−177.4 (3)	C17—C18—C19—C20	1.0 (6)
C4—C5—C6—C1	−0.9 (6)	C17—C18—C19—N4	−176.7 (3)
C5—C4—C7—C8	168.4 (4)	O3—N4—C19—C20	−5.6 (6)
C3—C4—C7—C8	−13.6 (6)	O4—N4—C19—C20	175.9 (4)
C4—C7—C8—C9	172.9 (4)	O3—N4—C19—C18	172.3 (5)
N2—N1—C9—C8	179.3 (3)	O4—N4—C19—C18	−6.3 (6)
N2—N1—C9—C10	3.5 (5)	C18—C19—C20—C21	−0.6 (6)
C7—C8—C9—N1	−171.1 (4)	N4—C19—C20—C21	177.2 (3)
C7—C8—C9—C10	4.9 (6)	C19—C20—C21—C16	−0.5 (5)
N1—C9—C10—C15	75.0 (5)	C19—C20—C21—N3	−178.2 (3)
C8—C9—C10—C15	−100.7 (4)	N2—C16—C21—C20	−177.4 (3)
N1—C9—C10—C11	−108.0 (4)	C17—C16—C21—C20	1.1 (5)
C8—C9—C10—C11	76.3 (5)	N2—C16—C21—N3	0.2 (5)
C15—C10—C11—C12	0.1 (6)	C17—C16—C21—N3	178.7 (3)
C9—C10—C11—C12	−176.9 (4)	O2—N3—C21—C20	1.9 (5)
C10—C11—C12—C13	1.4 (7)	O1—N3—C21—C20	−178.6 (3)
C11—C12—C13—F1	177.9 (4)	O2—N3—C21—C16	−175.8 (4)
C11—C12—C13—C14	−2.3 (8)	O1—N3—C21—C16	3.7 (5)
C12—C13—C14—C15	1.6 (7)		

### Hydrogen-bond geometry (Å, º)

**Table:**

*D*—H···*A*	*D*—H	H···*A*	*D*···*A*	*D*—H···*A*
N2—H21···O1	0.86	1.95	2.584 (4)	130
C11—H11···O4^i^	0.93	2.45	3.316 (7)	154

Symmetry code: (i) −*x*+1, −*y*+1, −*z*+1.
